# The Use of Bovine Colostrum in Medical Practice and Human Health: Current Evidence and Areas Requiring Further Examination

**DOI:** 10.3390/nu14010092

**Published:** 2021-12-27

**Authors:** Raymond John Playford

**Affiliations:** School of Biological Sciences, University of West London, St Mary’s Road Ealing, London W5 5RF, UK; Ray.Playford@uwl.ac.uk; Tel.: +44-(0)20-8579-5000

Colostrum is produced by the mammary gland for the first few days following birth and is a rich natural source of macro- and micro-nutrients, immunoglobulins, and peptides with anti-microbial, immune modulatory and/or growth-factor activity. Bovine colostrum (BC) is beneficial for the nutritional and immunological support, growth, and development of newborn calves. BC is produced in excess of that required by suckling calves, and this excess is collected, processed and sold commercially to promote general health and immune support for animal husbandry and for human use and also shows potential for treatment of several medical conditions. 

This Special Issue of *Nutrients*, “The Use of Bovine Colostrum in Medical Practice and Human Health” contains five review articles covering various aspects of the potential use of BC, including its main constituents and uses [1], use in gastrointestinal health and disease [2], sports nutrition [3], pediatrics [4], and immune support [5]. It also includes a systematic review of its clinical use [6] and an original research article examining its effects on enteric bacteria [7]. Taken together, they provide an excellent critique and extensive bibliography of the current state of knowledge and provide a series of challenges that need to be addressed by industry and researchers to fully understand the potential value of BC for human use. 

The article by Playford and Weiser [1] provides a detailed overview of the constituents of BC, including macro- and micro-nutrients, with a particular emphasis on components with potential bioactive relevance. BC is rich in immunoglobulins, particularly IgG, which is present in approx. 10-fold higher concentrations than mature milk. BC also contains multiple other factors that may affect immunity in humans, such as cytokines and lactoferrin. It is important to note a major difference between the suckling calf and the neonatal human; unlike the human situation, placental transfer of IgGs does not occur in the cow, meaning that early ingestion of BC to enhance passive immunity in the calf is probably of greater importance than in the human situation, where the baby is already born with circulating immunoglobulins derived from the mother’s blood. If humans ingest BC, the bovine IgGs within the lumen are available to bind pathogens (if the cow donor has been exposed to the antigen) [7] and may also induce some indirect immunological effects [1,5]. However, bovine IgG probably does not traverse the human gut barrier intact and will therefore not result in active bovine-derived IgGs being present in the recipient’s bloodstream (with the possible exception of premature human infants). Any generalized immune effect of BC in humans is therefore probably mediated by mechanisms and factors other than exclusively IgG-mediated direct antigen binding. 

BC is a rich source of growth factors, such as EGF and IGF-1, with concentrations falling rapidly during the first 2–3 days post calving [1,8]. Several growth-factor constituents of BC elicit pro-proliferative (growth) and protective effects when tested in cell lines and in in vitro gastrointestinal models, suggesting relevance for maintaining gut integrity or stimulating repair. The article by Chandwe and Kelly [2] discusses the evidence regarding the potential value of BC for gastrointestinal conditions and is also covered in the systematic clinical review by Guberti [6] and, in relation to the use of BC to maintain gut integrity of athletes, in the article by Davison [3]. At least on a theoretical basis, one would expect this application to be one of the most optimistic, as the constituents of BC are able to directly interact with luminal pathogens, influence the gut microbiome and allow growth factors and cytokines direct access to the intestinal mucosa. 

BC shows promise for the treatment and/or prevention of infectious diarrhea in adults and children. Several human studies in children and adults, usually performed in low-income countries, suggest that the use of hyperimmunized BC against specific pathogens has value in reducing diarrhea. A smaller number of studies has shown positive effects when using non-hyperimmunized BC, especially against rotavirus and *E. coli*-induced diarrhea. It is therefore relevant that even “non-hyperimmunized” BC contains IgG targeted against a wide variety of potential pathogens (albeit at a lower concentration) due to natural exposure. This aspect was highlighted in the research article by Playford and Marchbank [7], who demonstrated that non-hyperimmune BC can reduce the fall in in vitro gut integrity of gut monolayers exposed to a variety of bacterial enteric pathogens, including those commonly associated with small-bowel bacterial overgrowth, leading the authors to recommend progression to clinical trials. Pilot clinical trials also suggest that BC may be useful to reduce non-steroidal anti-inflammatory-drug (NSAID)-induced gut injury and for the treatment of inflammatory bowel disease [1,2]. 

For hormonal growth factors within BC to elicit effects beyond the gastrointestinal tract, they must survive luminal digestion and be absorbed intact into the bloodstream. There is little evidence that this occurs, and if it did, it could be considered a “double-edged sword”, as increasing growth-factor constituents in the bloodstream could raise concerns over safety. Discussion of this topic occurs in several of the reviews and has special relevance for the potential of BC to raise plasma IGF-1 levels in athletes, potentially resulting in sanctions from “anti-doping” regulatory authorities. This is discussed in detail by Davison [3], who concludes that while there remains a potential concern, most studies suggest that consumption of even high doses of BC does not increase plasma IGF-1. As IGF-1 has a molecular weight of 7.5 KDa, which is similar to that of many of the other growth-factor and hormonal constituents within BC (such as epidermal growth, EGF), it seems likely that these other growth factors do not enter the bloodstream intact. Davison also discusses the limited data supporting the effect of BC supplementation of enhancement of body composition and athletic performance but advises that additional studies are required before any conclusion can be reached. 

Sangild and co-workers reviewed the evidence of use of BC in pediatric nutrition and health [4] and included a description of the value of the premature pig model as a surrogate for the human condition. There are several theoretical reasons why BC might have value for conditions affecting the newborn, including the fact that the gut lumen is probably more permeable to passage of intact proteins than the adult human gut. Although positive results of BC for the prevention of NEC in the pig model have been shown, review of the limited clinical data examining the value of BC for human NEC appears less optimistic [2,3,4]. Additional studies are currently ongoing, and results are awaited with interest. BC supplementation is also included in some “growing-up” or “follow-up” milk formulas, and Sangild discusses the value of such an application in high-income and/or low-income countries [4].

The article by Ghosh and Iacucci covers the effects of BC on immunity, focusing on human studies [5]. Athletes are known to be at increased risk of upper respiratory tract infections (URTI) for reasons that are not completely understood. Both the Ghosh and Davison articles [3,5] review studies on the use of BC in athletes to reduce incidence and severity of URTIs. Several studies have reported positive results, despite there being no consistent change in ex vivo blood markers of immune modulation. Several of the articles in this special edition also discuss the evidence for the use of BC for URTIs in other (non-athlete) populations, with the Sangild article stating there is some evidence for a beneficial effect of BC supplementation in reducing incidence of URTI in healthy children, but these studies need to be interpreted with caution, as they often have methodological issues, such as using open, non-controlled prospective protocols. Even when RCTs have been performed, significant methodological issues may limit their interpretation. For example, a paper quoted in the Davison review reported that BC supplementation was three times more efficacious in preventing influenza than giving the subject influenza vaccination, in a study involving healthy adults [9]. However, influenza infection was based on self-reported symptoms rather than definitive immunological diagnosis, with 18/23 (78%) of the healthy controls suffering at least one episode of “flu” in a three-month period, whereas one would expect an annual risk of between 5 and 20%. Furthermore, the influenza-vaccinated group had no significant reduction in “flu” episodes or duration of symptoms compared to non-vaccinated, non-treated controls, suggesting that most symptom episodes captured were not due to influenza infection at all. Nevertheless, taking all the studies and reviews together, the use of BC to prevent viral URTI or to enhance immunity more generally shows promise. However, as both Ghosh and Guberti [5,6] state, additional well controlled placebo-controlled, randomized trials involving different age groups are required to establish clinical efficacy. 

Several of the reviews highlight apparent inconsistencies in results between studies, making interpretation difficult. A potential contributor to this problem is that the “quality/efficacy” of the BC used may vary between studies. Commercial producers of BC mainly focus on total protein and IgG content as their “quality” measure. There is some merit in this, as IgG levels, along with many of the growth factors, fall rapidly over the first few days after calving [1,8]. However, a recent study has shown that the bioactivity of different BC commercial products varies widely (6-fold), even though they report similar total protein and IgG content [8]. This may be partly due to differences in the collection time of the BC post calving, but that is probably not the major reason, as studies on serial samples from a cohort of recently calved animals showed bioactivity of BC fell approximately in proportion to protein content during this period, while differences in bioactivity of commercial samples remained, even when normalized for protein content [8]. Bioactivity is therefore probably also being affected by actions occurring post collection, either by the manufacturers or distributors during the processing or storage of product. Bioactivity of BC is highly sensitive to heat exposure [8], and differences in pasteurization techniques may provide a partial explanation. Manufacturers of BC-containing consumer products that undergo a heating/baking stage therefore need to be aware of the potential risk of loss of bioactivity (such as pro-reparative growth-factor activity or, in the case of IgG, antigen binding), even though the apparent concentration of IgG or individual growth-factor level appear unchanged. As a result of these findings, several authors suggest that bioassays demonstrating effects such as IgG antigen binding and/or proliferative activity should be performed before embarking on clinical trials, as they provide more clinically relevant data than quantitating potentially inactivated peptides and proteins by measuring total IgG or a particular growth-factor content. 

The optimal dose of BC for human use is uncertain. Many commercial providers market BC as a health-food supplement and recommend a dose of 500 mg–1 g/day for healthy adults. However, as highlighted in the Ghosh and Guberti articles [5,6], most studies examining effects of BC on athletes use daily doses of between 10 and 20 g/day, with similar doses being used to examine effects of BC on NSAID gut injury in human studies. Nevertheless, some clinical studies have shown positive effects when tested at these 500 mg–1 g levels, and there is virtually no high-quality data examining dose-response effects in clinical settings, as would be the norm for pharmaceutical products. Further studies are required to demonstrate what is an effective dose, which may vary depending on the condition being considered. 

Several articles in this series comment on the potential use of BC in combination with other agents, with a view to acting synergistically, e.g., combining BC with egg, probiotics, minerals or herbal products, and preliminary results are discussed in the relevant articles. An additional approach is to improve the biological stability of BC constituents while they transit the gut lumen, e.g., by adding individual proteins with protease-inhibitor activity, such as soya bean trypsin inhibitor, or food components that include them, such as raw soy flour. Combining raw soy flour with BC (10% wt/wt) reduced loss of pro-proliferative activity of BC by about 50% when incubated in digestive enzymes in vitro [10]. Interestingly, this combination was much less efficacious in preserving IgG antigen-binding activity against digestion, possibly due to IgGs being much larger proteins than the growth factor constituents. An in vivo model of distal colitis also showed improved efficacy of the soya/BC combination compared to BC alone, presumably due to increased delivery of reparative/growth factors to the colon. Clinicals studies are required to examine whether this translates to the human condition.

BC for human use is usually marketed as a health-food supplement, and for regulatory reasons, specific medical claims are not allowed. BC is therefore usually advertised using terms such as “to support immune or digestive health”. In the health-food (immune support, GI support) supplement market, BC is better evidenced than many of its competitors. BC has also undergone preliminary testing for a variety of medical conditions, with some early indicators showing promise. However, as stated in the conclusion of the Ghosh article, to be marketed as a pharmaceutical product, the BC industry needs to address the issue of quality control, taking into account the variable bioactivity of commercially produced BC, combined with the need to accumulate more evidence from well conducted clinical randomized control trials. 

## Data Availability

Articles are licensed under an open access Creative Commons CC BY 4.0 license, meaning that anyone may download and read the paper for free.

## Abbreviations

BC	bovine colostrum
DSS	dextran sodium sulphate
EGF	epidermal growth factor
Ig	immunoglobulin
IGF	insulin-like growth factor
NSAID	nonsteroidal anti-inflammatory drugs
URTIs	upper respiratory tract infections
